# Effect of low-dose dexmedetomidine to prolong spinal anesthesia in elderly patients: a prospective randomized controlled study

**DOI:** 10.1186/s12871-024-02815-z

**Published:** 2024-11-26

**Authors:** Lisa Sangkum, Sivaporn Termpornlert, Choosak Tunprasit, Chatchayapa Rathanasutthajohn, Rojnarin Komonhirun, Sasima Dusitkasem

**Affiliations:** Department of anesthesiology, Faculty of medicine Ramathibodi Hospital, Mahidol University, Bangkok, 10400 Thailand

**Keywords:** Dexmedetomidine, Intravenous, Spinal anesthesia, Elderly, Transurethral resection of the prostate

## Abstract

**Background:**

Spinal anesthesia for transurethral resection of the prostate (TURP) has a short duration, which poses challenges for postoperative pain management. The present study aimed to investigate the effects of intravenous (IV) dexmedetomidine at a dosage of 0.4 µg/kg in prolonging the duration of spinal anesthesia and minimizing postoperative pain in elderly patients undergoing TURP.

**Methods:**

This prospective randomized controlled trial enrolled 38 patients aged 60–80 years who underwent elective TURP with spinal anesthesia. The patients were randomly assigned to two treatment groups: Group D received IV 0.4 µg/kg dexmedetomidine, whereas Group C received IV normal saline after spinal anesthesia administration. The primary outcome was the time to 2-dermatome regression.

**Results:**

The 2-dermatome regression time was longer in Group D than in Group C (104.44 ± 16.97 min vs. 80.63 ± 15.59 min, *p* < 0.05). The peak sensory block levels were significantly higher in Group D [T7 (T6–T8)] than in Group C [T10 (T7–T10)] (*p* = 0.017). The incidence of hypotension and bradycardia and postoperative pain at 0, 6, 12, and 24 h were not different between two groups.

**Conclusion:**

Intravenous dexmedetomidine at a dosage of 0.4 µg/kg significantly prolongs the duration of spinal sensory blockade. Although postoperative analgesia was not different, it provided hemodynamic stability without increasing the side effects.

**Supplementary Information:**

The online version contains supplementary material available at 10.1186/s12871-024-02815-z.

## Introduction

Spinal anesthesia is commonly used in transurethral resection of the prostate (TURP) procedures. Although low-dose bupivacaine can regulate spinal block levels with minimal hemodynamic effects, its short duration of action may not be sufficient for adequate anesthesia during surgery and postoperative pain control. Therefore, various methods have been developed to improve its efficiency and prolong the duration of spinal anesthesia to alleviate postoperative pain and minimize the use of opioids, which may cause numerous side effects in the elderly.

Dexmedetomidine, a selective α-2 adrenoreceptor agonist, exhibits sedative, analgesic, and anxiolytic properties following intravenous administration. In 2010, Kaya et al. revealed that intravenous (IV) dexmedetomidine at a dosage of 0.5 µg/kg in patients undergoing TURP extended spinal anesthesia, delayed the first analgesic request, and reduced postoperative pain medication demand [1]. A subsequent systematic review and meta-analysis conducted by Abdullah et al. confirmed these outcomes, showing a significant extension in spinal anesthesia duration and delayed first analgesic requests with IV dexmedetomidine [2].

However, the administration of dexmedetomidine may lead to adverse effects and excessive sedation, particularly in geriatric patients. Ko et al. [3] investigated the IV dexmedetomidine dosage for sedating elderly patients during spinal anesthesia. The findings revealed that dosages exceeding 0.5 µg/kg can result in hemodynamic instability and excessive sedation. The ED95, ensuring adequate sedation within 20 min for elderly patients, was 0.4 µg/kg. Consequently, the recommended dosage for elderly patients falls within the range of 0.4–0.5 µg/kg [3].

Hence, considering that elderly patients tend to be more vulnerable to the adverse effects of sedation, the present study aimed to investigate the effects of applying low-dose dexmedetomidine at a dosage of 0.4 µg/kg on prolonging spinal anesthesia.

## Methods

### Participants

We included patients aged 60–80 years with an American Society of Anesthesiologists (ASA) physical status class I–III who were scheduled for elective TURP under spinal anesthesia from December 2022 to October 2023 in Ramathibodi Hospital. Each patient underwent cognitive evaluation using the Mini-Mental State Examination Thai version 2002 and provided written informed consent before participating in the study. Moreover, patients with the contraindications to spinal anesthesia, allergies to local anesthetics or dexmedetomidine, bradycardia [heart rate (HR) of < 50 beats per min (bpm)], neurological disorders, and severe hepatic or renal failure were excluded from the study.

### Study design

The patients were randomly assigned to two treatment groups using computer-generated randomization (block-of-4) in two parallel groups design with a 1:1 allocation ratio: Group D, which received IV dexmedetomidine at a dosage of 0.4 µg/kg, and Group C, which received IV normal saline after spinal anesthesia. The random allocation sequence was generated by a designated researcher not involved in participant enrollment or intervention assignment. Participants were enrolled by clinical staff under the supervision of the principal investigator. The study drugs were prepared with a total volume of 5 mL in 5 mL syringes by an anesthesiologist who was not involved in the study.

None of the patients received premedication. Standard monitors, including noninvasive blood pressure, electrocardiogram, and pulse oximeter, were attached and baseline values were recorded. Both the patient and the anesthesiologist were blinded to the treatment group. The patient was placed in the lateral position and subarachnoid block with 2.4 ml of 0.5% bupivacaine was performed in the L3–L4 interspace using a 27-gauge Quincke’s spinal needle. After intrathecal administration, the patient was placed in the lithotomy position. The level of sensory blockade was assessed with the loss of pinprick sensations every 1 min for the first 10 min and thereafter every 10 min during surgery. The highest sensory level was recorded. Thereafter study drug was administered over a 20-min period. 2-dermatome regression was defined as recovery time from the highest sensory block level.

The systolic blood pressure (SBP), diastolic blood pressure (DBP), heart rate (HR), and pulse oximetry were recorded every 3 min for the first 15 min following spinal anesthesia, and then every 5 min till the end of surgery. Intraoperative hypotension (SBP of < 90 mmHg or > 20% decrease from baseline) and bradycardia (HR of < 50 bpm) were treated with intravenous ephedrine (6 mg) and atropine (0.6) mg, respectively. The presence of hypotension, bradycardia, hypoxia, and adverse effects, such as nausea, shivering, vomiting, and pruritus, were recorded in the operating room and the postanesthesia care unit (PACU). Excessive sedation, defined as sluggish or no response to light glabellar tap or loud auditory stimulus (Ramsay Sedation Score > 4), was assessed every 10 min using a yes/no criterion. This assessment was accompanied by a pinprick stimulation test. The duration of surgery was noted. Postoperative pain, using the visual analog scale (VAS), at 0, 6, 12, and 24 h and the time to first analgesics were assessed and recorded by the staff nurse after surgery.

### Statistical analysis

The sample size was estimated based on a previous study by Jung et al. in 2013 [4], aiming for sufficient power to detect differences in 2-dermatome regression time. A sample size of 16 individuals per group was determined for a level of significance of 0.05 and power of 0.8. The sample size was increased by approximately 20% to allow for a possible dropout during the research process, resulting in a final participant count of 19 or 38 in total.

Data analysis was conducted using statistical software packages, namely, SPSS version 18.0 (SPSS Inc., Chicago, IL, USA) and STATA 16.0. Descriptive statistics, including mean, median, SD, and percentages, were reported as appropriate for the characteristics of the data. The continuous variables underwent distribution analysis, with the selection of either Student’s t-test or Mann-Whitney U test depending on the data’s distributional properties. The normality of continuous data was assessed using the Shapiro–Wilk test. For data deviating from normality, the median and interquartile range (25th and 75th percentiles) were computed, and nonparametric tests were performed. Categorical variables were analyzed using either the chi-square test or Fisher’s exact test, as appropriate. Statistical significance was set at a *p-value* of < 0.05. Intention-to-treat analysis provided a robust evaluation of treatment efficacy, ensuring a comprehensive understanding of the intervention’s impact.

## Results

### Baseline demographic and clinical characteristics

**Fig. 1:**
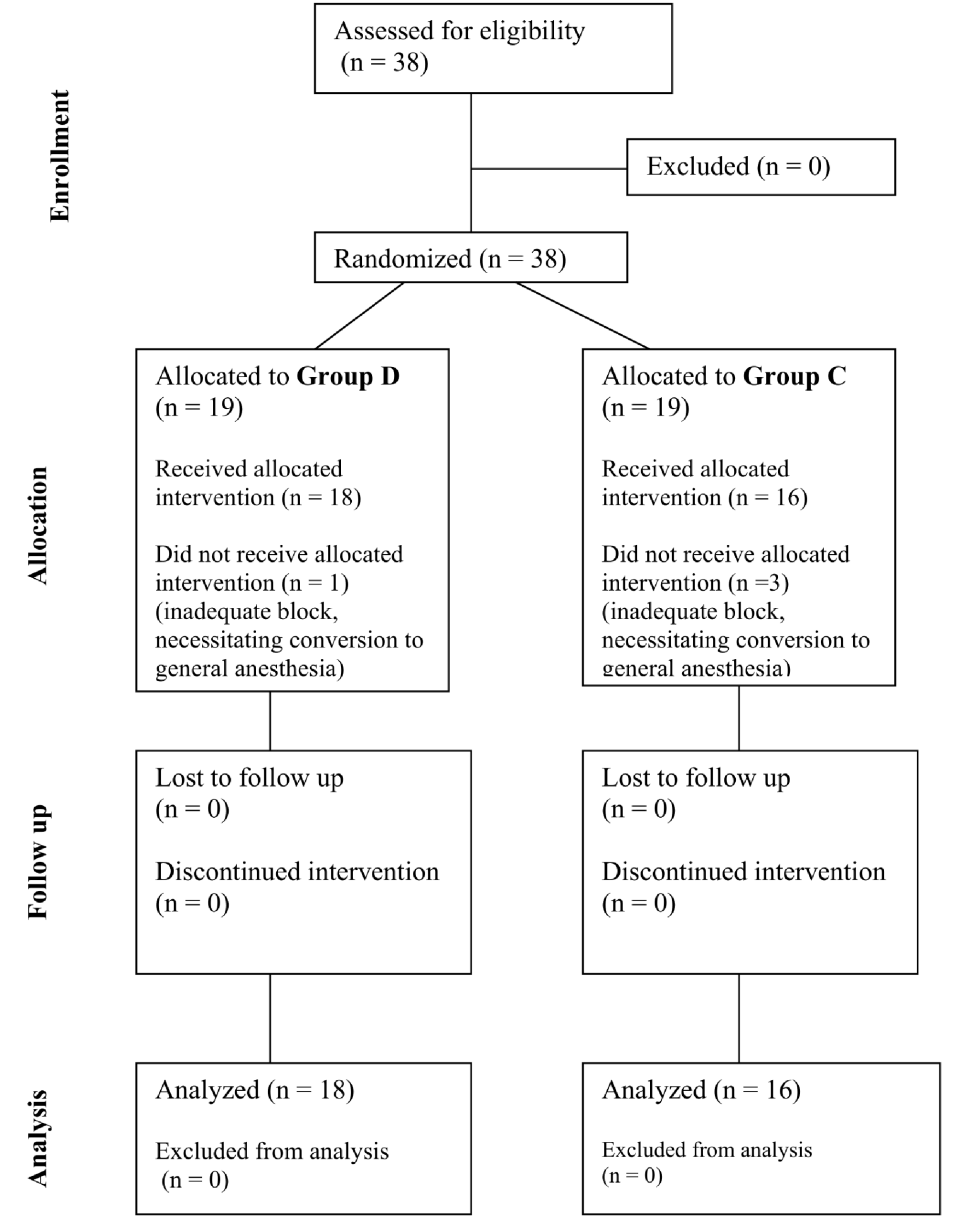
Participant flow

A total of 38 patients were enrolled in the present study from December 2022 to October 2023. Patients were randomized to Group D or Group C after spinal anesthesia using an equal allocation. One patient from Group D and three patients from Group C were excluded due to inadequate block, which requires the conversion to general anesthesia (Fig. 1: See supplementary). Thirty-four remaining patients were then included. The demographic characteristics are presented in Table 1.

**Table 1:** Demographic data

	Group D (18)	Group C (16)
Age (year)	69.28 ± 6.54	71.88 ± 6.29
Body mass index (kg.m^− 2^)	24.66 ± 4.10	24.08 ± 4.00
ASA physical status		
II	10 (55.56%)	3 (18.75%)
III	8 (44.44%)	13 (81.25%)
Operation time (min)	96.67 ± 44.89	104.38 ± 39.49

Data are expressed as mean ± standard deviation or n (%)

Abbreviations: ASA, American Society of Anesthesiologists

**Fig. 2:**
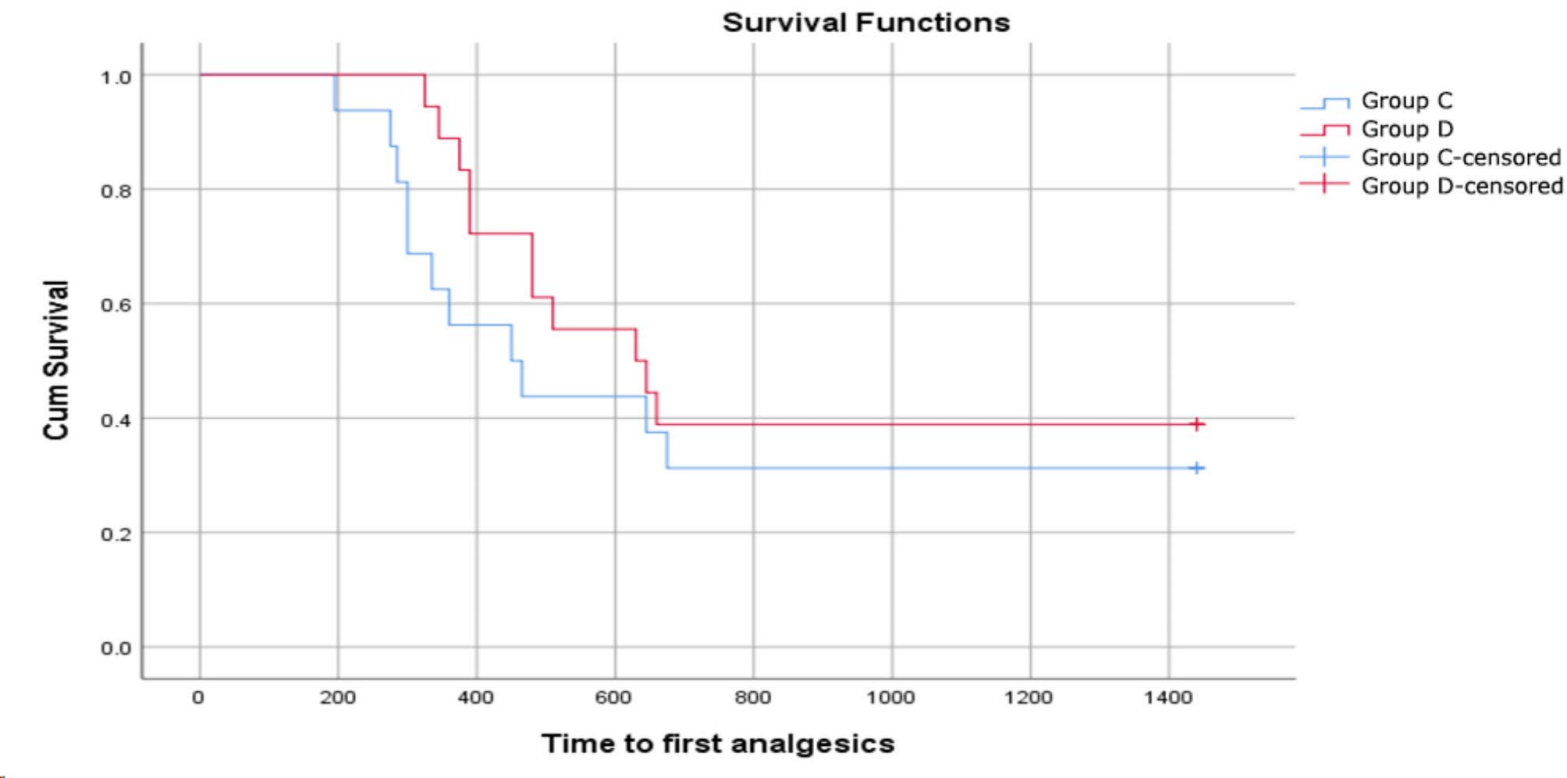
Survival function of time to administration of the first analgesic

The peak sensory block levels were significantly higher in Group D [T7 (T6–T8)] than in Group C [T10 (T7–T10)] (*p* = 0.017). The 2-dermatome regression time was longer in Group D than in Group C (104.44 ± 16.97 vs. 80.63 ± 15.59 min, *p* < 0.05). The incidences of hypotension and bradycardia were not significantly different between the two groups (Table 2). Moreover, postoperative pain at 0, 6, 12, and 24 h (Table 3) and the time to first analgesic (Fig. 2: See supplementary) were not significantly different between the two groups (*p* = 0.355). Overall, no complications, such as excessive sedation, hypoxia, nausea/vomiting, or shivering, were reported.

**Table 2:** Intraoperative data

	Group D (18)	Group C (16)	*p*-value
Peak level of block	T7 (T6–T8)	T10 (T7–T10)	0.017
2-Dermatome regression time (min)	104.44 ± 16.97	80.63 ± 15.59	0.001*
Hypotension	5 (27.78%)	2 (12.50%)	0.405
Bradycardia	8 (44.44%)	2 (12.50%)	0.063

Data are expressed as mean ± standard deviation, median (25th and 75th percentiles), or n (%)

**Table 3:** Postoperative visual analog pain scale

	Group D (18)	Group C (16)	*p*-value
Visual Analog Pain Scale 0–10			0.144
0 h	0 (0–0)	0 (0–0)	
6 h	3 (3–5)	5 (3.5–7.5)	
12 h	2 (0–3)	3 (0.5–3.5)	
24 h	0 (0–0)	0 (0–0)	

Data are expressed as median (25th and 75th percentiles)

Multivariable linear regression adjusting for ASA classification and peak block height revealed that the prolonged 2-dermatome regression time remained significant (*p* = 0.006).

The multivariable logistic regression for hypotension and bradycardia also showed no significant differences (See supplementary table).

## Discussion

In this randomized double-blind placebo-controlled clinical study, a significant prolongation of spinal anesthesia was observed in Group D. The duration of 2-dermatome regression time in Group D was 104.44 ± 16.97 min compared with 80.63 ± 15.59 min in Group C (*p* < 0.05). Although no significant differences in postoperative pain levels were observed, the overall postoperative pain experienced by patients in Group D was comparatively lower than that in Group C.

Dexmedetomidine, a selective α-2 adrenoreceptor agonist, exhibits sedative, analgesic, and anxiolytic properties following intravenous administration. Several studies have shown a preferable efficacy of dexmedetomidine in prolonging the duration of spinal anesthesia compared with other sedative medications, including intravenous midazolam [1, 5], propofol [6], and clonidine [9, 10]. Furthermore, dexmedetomidine sedation leads to a lower incidence of delirium, which demonstrated a beneficial effect on postoperative recovery in elderly patients [7, 8]. Most trials typically included initial loading doses of dexmedetomidine ranging from 0.5 to 1 µg/kg with a maintenance infusion [1, 2, 4–6, 9–15]. Nevertheless, although the effectiveness of dexmedetomidine increased with increasing dose, higher incidences of hypotension and excessive sedation have been found [16, 17]. Moreover, elderly patients tend to be more vulnerable to adverse effects and may require lower doses of dexmedetomidine. In the setting of anesthesia, Kuang et al. [18] showed a declining clearance of dexmedetomidine and more patients requiring intervention in the elderly group compared with the young group (68.75% vs. 36.84%). However, Park et al. [19] found that excessive sedation occurred in 46% of elderly patients receiving a relatively small dose of dexmedetomidine (0.5 µg/kg). Ko et al. [3] also reported a similar trend and recommended a dosage of 0.4–0.5 µg/kg in geriatric patients during spinal anesthesia to minimize the risk of excessive sedation and hemodynamic instability.

The present study showed a significant prolongation of the regression of two dermatomes with single doses of dexmedetomidine (0.4 µg/kg) without increasing the incidence of bradycardia and hypotension. These results were similar to a study conducted by Jung et al. [4], which reported the effectiveness of small, single-dose intravenous dexmedetomidine (0.25–0.5 µg/kg) in improving the duration of spinal anesthesia and providing hemodynamic stability. From our study, no excessive sedation or serious respiratory complications were reported based on intraoperative or PACU data. This is consistent with the findings of a previous study by Jeongmin et al. [20], which suggested that the dexmedetomidine dosage for elderly patients should be reduced to two-thirds of the dose for young patients to avoid over-sedation and the ED95 for light sedation was 0.38 µg/kg in elderly patients undergoing neuraxial blockade.

While the lower dose of dexmedetomidine (0.4 µg/kg) was sufficient to prolong spinal anesthesia, duration compared to the control group, it did not enhance postoperative analgesia as significantly as in other studies with higher doses [1, 2, 4–6, 9–15]. Additionally, the low postoperative pain scores reported in both groups could be due to other factors, such as postoperative care or the inherently low pain associated with the surgical procedure, rather than the dosage of dexmedetomidine itself.

This study has some limitations. First, the sample size was relatively small, which may have limited the power to detect statistically significant differences, particularly for secondary outcomes like postoperative pain and adverse events. Second, there was a baseline imbalance in ASA physical status between the groups, with more ASA III patients in the control group. Although the prolonged 2-dermatome regression time remained significant after using multivariable linear regression, other outcomes (e.g., Peak level of block and postoperative pain) may also have been affected by the baseline differences in ASA.

## Conclusion

A single dose of intravenous dexmedetomidine (0.4 µg/kg) effectively prolongs the duration of spinal anesthesia in elderly patients undergoing TURP. It provides hemodynamic stability without increasing the side effects. However, its benefits in enhancing postoperative analgesia were not significant.

## Electronic supplementary material

Below is the link to the electronic supplementary material.

Supplementary Material 1

Supplementary Material 2

## Data Availability

Availability of data and materials: The datasets used and/or analysed during the current study are available from the corresponding author on reasonable request.
