# Supplementary material for: Effect of low-dose dexmedetomidine to prolong spinal anesthesia in elderly patients: a prospective randomized controlled study
Source: BMC Anesthesiol. 2024 Nov 26;24:427. doi: 10.1186/s12871-024-02815-z (PMC11590567; doi:10.1186/s12871-024-02815-z)
Supplement: Supplementary file 2 — Supplementary Material 2 [file 12871_2024_2815_MOESM2_ESM.docx]

**Supplementary**

Multivariable regression model showing the association between dexmedetomidine group and significant outcomes of this study, adjusting for ASA physical status classification and peak block height

|  | **Group D** (18) | **Group C** (16) | ***p*-value** |
| --- | --- | --- | --- |
| Hypotension | 5 (27.78%) | 2 (12.50%) | 0.809 |
| Bradycardia | 8 (44.44%) | 2 (12.50%) | 0.273 |
|  |  |  |  |
| 2-dermatome regression time (min) | 104.44 ± 16.97 | 80.63 ± 15.59 | 0.006* |

Data are mean ± standard deviation, or n (%).

*Statistically significant at p<0.05
